# LuxS-independent formation of AI-2 from ribulose-5-phosphate

**DOI:** 10.1186/1471-2180-8-98

**Published:** 2008-06-18

**Authors:** Timothy J Tavender, Nigel M Halliday, Kim R Hardie, Klaus Winzer

**Affiliations:** 1 Institute of Infections, Immunity, and Inflammation, University of Nottingham, Centre for Biomolecular Sciences, Nottingham, NG7 2RD, UK; 2 School for Health and Medicine, Division of Biomedical and Life Sciences, Lancaster University, Bailrigg, Lancaster, LA1 4YQ, UK; 3 Faculty of Life Sciences, Michael Smith Building, University of Manchester, Manchester, M13 9PT, UK

## Abstract

**Background:**

In many bacteria, the signal molecule AI-2 is generated from its precursor *S*-ribosyl-L-homocysteine in a reaction catalysed by the enzyme LuxS. However, generation of AI-2-like activity has also been reported for organisms lacking the *luxS *gene and the existence of alternative pathways for AI-2 formation in *Escherichia coli *has recently been predicted by stochastic modelling. Here, we investigate the possibility that spontaneous conversion of ribulose-5-phosphate could be responsible for AI-2 generation in the absence of *luxS*.

**Results:**

Buffered solutions of ribulose-5-phosphate, but not ribose-5-phosphate, were found to contain high levels of AI-2 activity following incubation at concentrations similar to those reported *in vivo*. To test whether this process contributes to AI-2 formation by bacterial cells *in vivo*, an improved *Vibrio harveyi *bioassay was used. In agreement with previous studies, culture supernatants of *E. coli *and *Staphylococcus aureus luxS *mutants were found not to contain detectable levels of AI-2 activity. However, low activities were detected in an *E. coli pgi-eda-edd-luxS *mutant, a strain which degrades glucose entirely via the oxidative pentose phosphate pathway, with ribulose-5-phosphate as an obligatory intermediate.

**Conclusion:**

Our results suggest that LuxS-independent formation of AI-2, via spontaneous conversion of ribulose-5-phosphate, may indeed occur *in vivo*. It does not contribute to AI-2 formation in wildtype *E. coli *and *S. aureus *under the conditions tested, but may be responsible for the AI-2-like activities reported for other organisms lacking the *luxS *gene.

## Background

In the marine bacterium *Vibrio harveyi*, autoinducer 2 (AI-2) is one of three quorum-sensing molecules regulating the production of bioluminescence in a population-density-dependent fashion [1,2]. In recent years, numerous pathogenic and non-pathogenic bacteria have also been shown to produce AI-2 (for a recent review see [3]), and for this reason the molecule has been suggested to function in interspecies communication [1,4-7].

**Figure 1:**
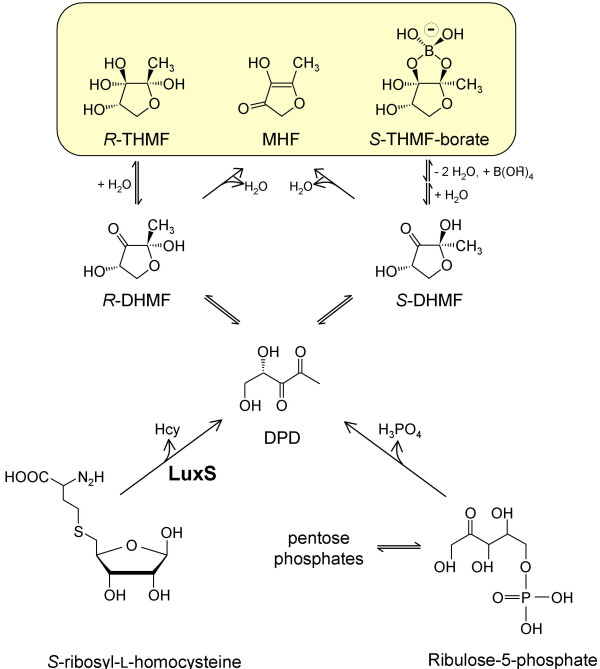
**Pathways of DPD and AI-2 formation**. The schematic integrates pathways described for production of 4,5-dihydroxy-2,3-pentanedione (DPD) from D-ribulose-5-phosphate [13] and *S*-ribosylhomocysteine (SRH) [4] with the subsequent formation of AI-2 molecules (yellow box) detected by *V. harveyi *and *S. enterica *serovar Typhimurium [10]. Ribulose-5-phosphate is formed enzymatically from other sugar phosphates and its reactive open-chain carbonyl form in aqueous solution facilitates DPD generation. DPD cyclisation leads to several products forming via 2,4-dihydroxy-2-methylhydrofuran-3-one intermediates, including two distinct autoinducer molecules, *S*-THMF-borate ((2*S*,4*S*)-2-methyl-2,3,3,4-tetrahydroxytetrahydrofuran-borate; detected by *V. harveyi*) and *R*-THMF ((2*R*,4*S*)-2-methyl-2,3,3,4-tetrahydroxytetrahydrofuran; detected by *S. enterica *serovar Typhimurium) as well as MHF (4-hydroxy-5-methyl-3(2H)-furanone). Intermediates or side products shown are: *S*-THMF, (2*S*,4*S*)-2-methyl-2,3,3,4-tetrahydroxytetrahydrofuran; *R*-DHMF, (2*R*,4*S*)-2,4-dihydroxy-2-methyldihydrofuran-3-one; *S*-DHMF: (2*S*,4*S*)-2,4-dihydroxy-2-methyldihydrofuran-3-one; Hcy, homocysteine.

AI-2 is the collective term for a group of signal molecules formed from a common precursor, 4,5-dihydroxy-2,3-pentanedione (DPD). DPD is generated by many bacteria as a by-product of the activated methyl cycle in a reaction catalysed by LuxS [4,8]. LuxS acts by cleaving *S*-ribosylhomocysteine (SRH) to yield homocysteine and the reactive DPD, which spontaneously cyclises to from a range of furanone derivatives (Fig. 1). Two of these, (2*S*,4*S*)-2-methyl-2,3,3,4-tetrahydroxytetrahydrofuran-borate (*S*-THMF-borate) and (2*R*,4*S*)-2-methyl-2,3,3,4-tetrahydroxytetrahydrofuran (*R*-THMF), are recognised by specific periplasmic binding proteins in *V. harveyi *and *Salmonella enterica *Serovar Typhimurium, respectively [9,10]. Another derivative, 4-hydroxy-5-methyl-3(*2H*)-furanone (MHF), has also been confirmed as a product of the LuxS catalysed reaction *in vitro *[8] and shown to have moderate bioluminescence inducing ability in *V. harveyi *[4,8].

Interestingly, formation of MHF from D-ribulose-5-phosphate (Rul-5-P) has also been reported. This phenomenon was initially witnessed following the action of spinach phosphoriboisomerase on ribose-5-phosphate (Rib-5-P) but was dismissed as an anomalous side-activity of the enzyme [11]. The authors demonstrated a transient accumulation of an unknown intermediate, formed from the sugar phosphate, which subsequently converted to MHF. Later, Hauck *et al*. demonstrated that MHF arose spontaneously from Rul-5-P, and identified the unknown intermediate as DPD [12,13]. Thus, spontaneous conversion of Rul-5-P may provide a novel route by which AI-2 could form in a *luxS*-independent fashion. However, whilst it is known that Rul-5-P gives rise to DPD under certain conditions *in vitro*, and that chemically synthesised DPD can stimulate bioluminescence in *V. harveyi *AI-2-responsive reporter strains [14-16] the principle of LuxS-independent formation of AI-2 activity from Rul-5-P in biologically relevant quantities has yet to be proved. Furthermore, the possibility and the ramifications of this process occurring *in vivo *have not been previously addressed. Here we demonstrate that spontaneous conversion of Rul-5-P does indeed give rise to potent AI-2 activity and investigate various *luxS *mutants, including an *E. coli *strain affected in central carbon metabolism, for LuxS-independent AI-2 formation *in vivo*. In addition, consideration is given to the possible implications of this alternative mechanism for AI-2 production.

## Results and discussion

### High levels of AI-2 activity arises from ribulose-5-phosphate *in vitro*

**Figure 2:**
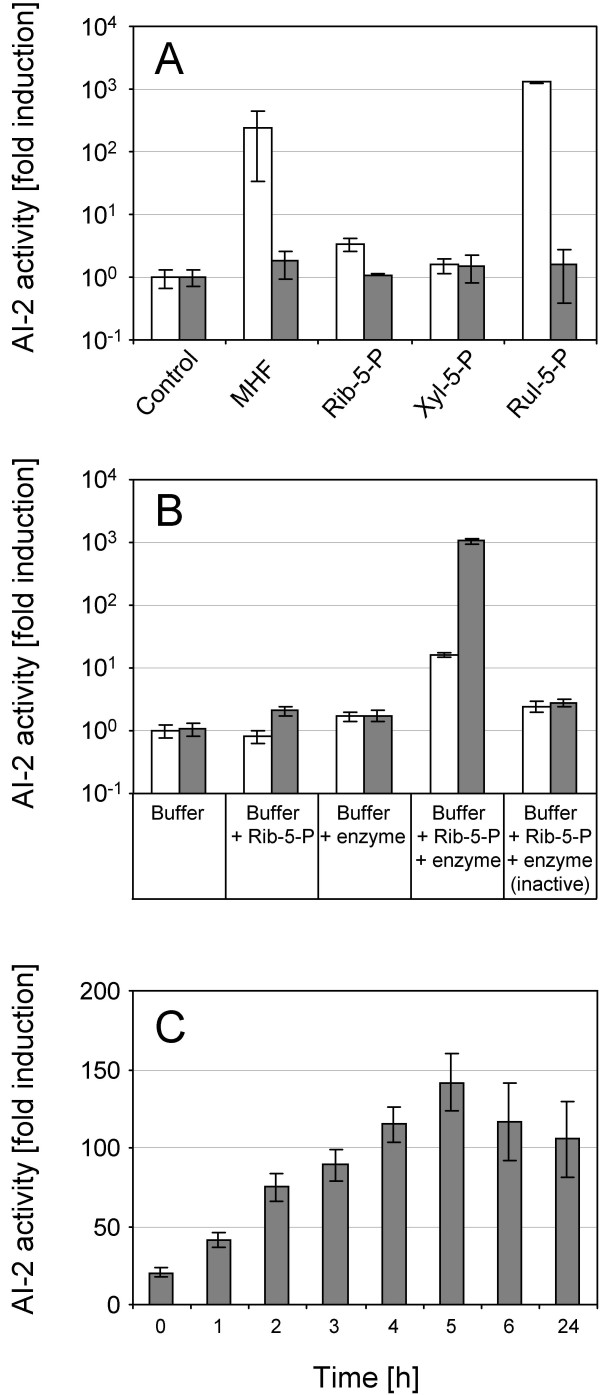
**Spontaneous generation of AI-2 activity in ribulose-5-phosphate solutions**. **(A) **AI-2 activity in pentose phosphate solutions. Reaction buffer (10 mM sodium phosphate, pH 7.7) was incubated alone (control) or containing either 5 mM MHF, 5 mM ribose-5-phosphate (Rib-5-P), 5 mM xylulose-5-phosphate (Xyl-5-P), or 5 mM ribulose-5-phosphate (Rul-5-P). After incubation for 24 h at 37°C, the solutions were analysed for the presence of bioluminescence-inducing activity using the *V. harveyi *BB170 and BB886 bioassays. *V. harveyi *BB170 (white bars) is specifically activated by AI-2 and BB886 (grey bars) by AI-1. **(B) **AI-2 activity in 5 mM ribose-5-phosphate solutions incubated with phosphoriboisomerase (10 U/ml). Samples were taken immediately after the start of the experiment (white bars, 0 h) or after 2 h incubation at 37°C (grey bars). Controls contained reaction buffer only (buffer), no enzyme (buffer + Rib-5-P), no substrate (buffer + enzyme), or heat-inactivated enzyme (buffer + Rib-5-P + enzyme (inactive)). **(C) **Kinetics of AI-2 formation in Rul-5-P solutions. 0.5 mM Rul-5-P in reaction buffer was incubated at 37°C and samples removed and snap-frozen in liquid nitrogen at the times indicated. The samples were then analysed for AI-2 activity using *V. harveyi *BB170. Results represent the mean (± SD) of three independent experiments. AI-2 activity is determined by the fold-induction of light emission relative to that of the negative reaction buffer control. For comparison, approximately 400-fold induction was observed with *E. coli *MG1655 culture supernatants.

A solution of 5 mM Rul-5-P was incubated at 37°C for 24 h, based on the methods previously described for MHF formation [12,13], and assayed for bioluminescence-inducing activity in *V. harveyi *BB170, a bioreporter for detection of AI-2 activity [17]. Addition of this solution to the reporter strain induced high levels of bioluminescence, much higher than those observed for a positive control of 5 mM MHF. By comparison, the isomers Rib-5-P and xylulose-5-phosphate (Xyl-5-P) stimulated little or no bioluminescence (Fig. 2A). Since bioluminescence in *V. harveyi *is also controlled by the signal molecule *N*-3-hydroxybutanoyl-L-homoserine lactone (AI-1), each compound was also tested with the AI-1 responsive bioreporter, *V. harveyi *BB886 [17]. No bioluminescence was induced in this strain (Fig. 2A) indicating Rul-5-P-mediated bioluminescence to be induced specifically via the AI-2 detection system.

To demonstrate that the observed AI-2 activity was not caused by impurities present in the commercial preparations, Rul-5-P was also produced enzymatically. Incubation of 5 mM ribose-5-phosphate in the presence of active spinach phosphoribose isomerase (10 U/ml), which isomerised the substrate to Rul-5-P, resulted in the formation of high AI-2 activity, whereas no significant activity was observed after incubation with heat-inactivated enzyme (Fig. 2B). Furthermore, time course experiments performed with freshly prepared 0.5 mM Rul-5-P incubated at 37°C showed that AI-2 activity gradually increased over time, typically displaying a maximum after 5 hours (Fig. 2C). This was consistent with a conversion of Rul-5-P firstly to DPD, and then to the less-active MHF, in agreement with previously observed spectrophotometric data [11,13]. Fig. 2C shows that approximately 20-fold induction of bioluminescence was observed for the sample removed at the beginning of the time course experiment (0 h). This activity most likely originated from Rul-5-P conversion during the several hours of incubation in the *V. harveyi *BB170 bioassay rather than an intrinsic activity of the compound itself.

The concentration range of Rul-5-P used in these experiments was chosen because it matches the intracellular concentrations reported for some organisms in the literature, such as yeast (approximately 0.5 mM; [18]). However, much lower values in the range of 1–10 μM can be estimated from metabolite determinations in animal and human tissues, including liver, brain, blood samples [19-22].

Rul-5-P has been previously reported to give rise to MHF with approximately 1.3% of the sugar-phosphate converting to the furanone after incubation at pH 7.5 and 35°C for 15 h [13]. Liquid chromatography/tandem mass spectrometry (LC-MS/MS) confirmed that MHF was also formed under the conditions used in this study (data not shown), with conversion rates below 1% after 24 h of incubation at pH 7.7 and 37°C. Taking into account the low conversion rates, much higher AI-2 activities were observed in Rul-5-P solutions than could be expected solely due to formation of MHF (Fig. 2A and data not shown). This demonstrates that another compound derived from Rul-5-P, but different from MHF, was responsible for the observed activities. Since DPD is known to be generated in Rul-5-P solutions [13] and was also identified after *o*-phenylenediamine derivatisation under the incubation conditions used in this study (data not shown), this was most likely *S*-THMF-borate (see Fig. 1), a DPD-derivative known to activate the BB170 bioreporter [9].

### Modification of the AI-2 bioassay

**Figure 3:**
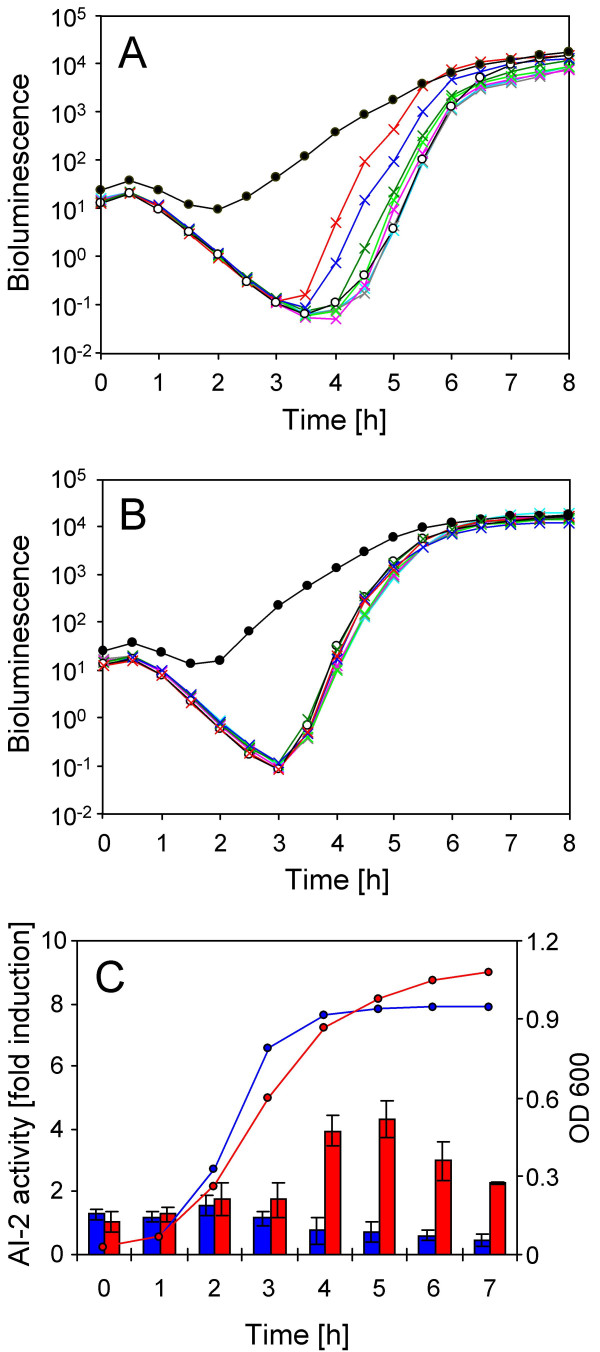
***E. coli *MG1655 pgi-EDP *luxS *produces extracellular AI-2 activity**. **(A) **Conventional *V. harveyi *BB170 bioassay [17] with *E. coli *DH5α culture supernatants obtained from cultures growing in LB + 0.5% (w/v) glucose. Turquoise, grey, pink, bright green, dark green, blue, and red lines indicate the bioluminescence observed for *E. coli *DH5α culture supernatants after 0, 1, 2, 3, 4, 5, and 6 h of growth, respectively. Open circles, negative control (LB medium + 0.5% glucose); closed circles, AI-2 containing positive control (*E. coli *MG1655 culture supernatant after 3 h of growth). **(B) **Modified *V. harveyi *BB170 bioassay with the same *E. coli *DH5α culture supernatants as analysed in (A). 25 mM HEPES was present in the bioassay medium to prevent acidification. For figure legends, see (A). **(C) **Growth (lines) and AI-2 activity profiles (bars) for *E. coli *MG1655 *luxS *(blue) and *E. coli *MG1655 *pgi*-EDP *luxS *(red). Each strain was grown in LB medium + 0.5% (w/v) glucose and samples removed hourly. For each sample the optical density at 600 nm was recorded and AI-2 activity in culture supernatants recorded using the modified *V. harveyi *BB170 bioassay containing 25 mM HEPES. Results represent the mean of three independent bioassays. For AI-2 activity, error bars represent the standard deviations. The experiments were repeated five times with similar results.

To determine whether LuxS-independent production of AI-2 also occurs *in vivo, E. coli *DH5α (which contains a frameshift mutation in *luxS*) and *Staphylococcus aureus *Newman *luxS *were each cultured in LB medium in the presence of 0.5% glucose.  In *E. coli*, glucose at this concentration had previously been shown to prevent AI-2 uptake via catabolite repression [23]. In each case, cell-free culture supernatants from mid- to late exponential phase onwards appeared to display 3–6 fold greater induction of bioluminescence in *V. harveyi *BB170 than controls of sterile control medium (not shown). However, following careful scrutiny it became apparent that this induction was not caused by true AI-2 activity. Metabolism of glucose led to acidification of the culture environment, most prominently between 3–5 hours of growth, during which time pH values typically declined from pH 7 to pH 5. Addition of culture-supernatants from this period onwards, to the *V. harveyi *BB170 bioassay, led to a subsequent decrease in pH of the bioassay medium (not shown). This coincided with enhanced recovery of endogenously-induced bioluminescence in *V. harveyi *BB170, following dilution into the assay medium (Fig. 3A). Acidification has been previously reported by DeKeersmaecker and Vanderleyden [24] to impact on the AI-2 bioassay, therefore, the assay medium was modified to include a final concentration of 25 mM HEPES buffer to increase the total buffer strength. This prevented the more acidic culture-fluids from influencing the pH of the assay medium and simultaneously eliminated the induction of bioluminescence observed for the *luxS *mutant culture supernatants (Fig. 3B). Incorporation of HEPES did not affect the detection of true AI-2 activity in culture-fluid samples as shown by the unaffected response to positive control samples. Overall, the data demonstrated that culture-supernatants of the two *luxS *mutants grown in LB medium did not contain detectable AI-2 activity.

### AI-2 activity is detectable in an *E. coli luxS *mutant with altered carbon flow

AI-2 activity was not apparent in the culture-fluids of *luxS *mutants investigated above. However, in *E. coli *at least, just 25% of exogenously supplied glucose is believed to be catabolised via the oxidative pentose phosphate pathway (OPPP) to yield the intermediate Rul-5-P [25]. It is therefore possible that LuxS-independent generation of AI-2 from Rul-5-P may be limited under the growth condition used in this study. It is also possible, that Rul-5-P concentrations are generally very low in *E. coli *and related organisms. In order to maximise the potential for detectable AI-2 production from the sugar-phosphate *in vivo*, an *E. coli *MG1655 *pgi*, *edd*, *eda *triple mutant (*E. coli *pgi-EDP; [25]) was utilised. In this strain, glucose catabolism occurs exclusively via the OPPP as entry of the sugar into the glycolytic and Entner-Doudoroff pathways are blocked ([25]; *pgi *encodes phosphoglucose isomerase; *edd *and *eda *encode phosphogluconate dehydratase and 2-keto-3-deoxy-6-phosphogluconate aldolase, respectively). Production of LuxS-derived AI-2 activity by *E. coli *pgi-EDP was eliminated through the introduction of a *luxS *mutation. This was achieved by P1 bacteriophage transduction of the disrupted *luxS *gene from *E. coli *BL21 *luxS *[9] into *E. coli *pgi-EDP. The mutation was also introduced into the *E. coli *MG1655 parent strain.

*E. coli *MG1655 *luxS *and pgi-EDP *luxS *were grown in LB containing 0.5% glucose. Cell-free culture-supernatants were harvested and tested for bioluminescence-inducing ability using the optimised (HEPES-containing) *V. harveyi *BB170 bioassay. *E. coli *pgi-EDP *luxS *culture-supernatants induced a response in *V. harveyi *BB170 that, whilst modest, was clearly not observed for *E. coli *MG1655 *luxS *(Fig. 3C). Typically a peak in bioluminescence-inducing activity was observed in *E. coli *pgi-EDP *luxS *culture-fluids following 5 hours of culture and displayed 4–6 fold greater bioluminescence induction than sterile LB alone. However, in the absence of glucose, AI-2 activity could not be detected (data not shown).

Several potential sources of false-positive results were investigated to determine whether the observed effects were a result of some non-AI-2-specific influence as witnessed for the unmodified bioassay. Assay medium pH was recorded following addition of all test samples and verified as being unaffected, thus confirming that elevated bioluminescence-induction by *E. coli *pgi-EDP *luxS *culture-fluids was not an artefact of acidification of the culture medium. In addition, viable counts were performed for the *V. harveyi *BB170 assay samples used to generate the data in Fig. 3C to ensure that differences in bioluminescence-induction between supernatant samples were not caused by effects on proliferation of the reporter strain (not shown). Furthermore, the possibility of obtaining erroneous results due to the repressive effects of glucose upon bioluminescence, as described by [24], was eliminated through the use of AI-2-negative media control samples with and without added glucose. These data therefore indicated that *E. coli *pgi-EDP *luxS *culture-fluids contained very low but detectable levels of AI-2 activity. This suggests that increased flux through the OPPP may indeed lead to increased cellular generation of AI-2-like molecules via the DPD intermediate. However, whether the OPPP represents one of the alternative major AI-2 production pathways predicted to exist in *E. coli *by stochastic modelling [26] remains doubtful, as only very low amounts of AI-2 could be detected for a metabolically crippled *E. coli *MG1655 *pgi*-*edd*-*eda-luxS *quadruple mutant but not *E. coli *MG1655 *luxS*. Future work will require the development of more stringent physical or chemical methods to unequivocally detect and quantify AI-2 molecules in complex biological samples, particularly when present at low concentrations. Once developed, such methods may allow more robust quantitative detection of, and distinction between, different DPD-derivatives. This would allow conclusive confirmation as to whether the Rul-5-P-dependent mechanism of AI-2 production is operational *in vivo*. An overview of the different pathways leading to DPD and AI-2 formation is given in Fig. 1

### Implications

Our finding that the DPD levels formed during the spontaneous conversion of Rul-5-P are sufficient to give rise to measurable AI-2 activity has several important implications. First, given the ubiquitous presence of Rul-5-P in metabolically active cells [27], it seems possible that molecules with AI-2 activity are intrinsically formed as by-products of pentose phosphate metabolism in all organisms and independently of the LuxS enzyme. Indeed, AI-2 activity has been reported in stationary phase culture-supernatants of *Streptococcus pyogenes luxS *mutants [28]. Furthermore, some microorganisms, plants, and animals are known to produce MHF [29-33], a compound which, at least in the case of certain yeasts, is believed to be derived from Rul-5-P [12,13].

Production of AI-2-like activity by higher organisms has also been demonstrated for algae of the genera *Chlamydomonas *and *Chlorella *[34] and evidence for the formation of DPD from Rul-5-P in tomato fruits has been provided [13]. Thus, organisms other than bacteria may have developed the machinery necessary to either metabolise or exclude DPD-derived by-products such as AI-2. This may also explain the presence of *lsr*-type AI-2 uptake systems in bacteria that do not contain a luxS homologue [3]. *Sinorhizobium meliloti*, for instance, possesses a complete putative *lsr *AI-2 uptake system and an AI-2 kinase (locus tags SMb21016-21022). Systems like this may have been acquired to minimise the loss of intrinsically produced DPD-derived compounds, or alternatively to scavenge the molecules released from other organisms present in the same niche, either bacteria or plants.

Finally, given the relative ease with which DPD, and thus AI-2, appears to form from Rul-5-P, and the potential advantages currently believed to be gained by the utilisation of AI-2-dependent signalling, it would seem an interesting, but as yet unexplored, possibility that some organisms may have acquired enzymes to direct and enhance this process of AI-2 formation in a controlled fashion.

## Conclusion

In conclusion, we have demonstrated that spontaneous degradation of Rul-5-P gives rise to substantial amounts of AI-2 activity *in vitro*. However, our data suggest that whilst this route may also be operational *in vivo*, in *E. coli *its contribution to AI-2 production is negligible. It could, however, be responsible for the AI-2-like signals reported for some higher organisms or bacteria lacking *luxS*. Given the postulated importance of AI-2-based quorum sensing in many pathogenic bacteria, the generation of DPD via a LuxS-independent route may have important implications.

## Methods

### Strains and media

*E. coli *DH 5α, *E. coli *BL21 *luxS*, *E. coli *MG1655 derivatives, and *S. aureus *Newman *luxS *[35] were routinely grown in Luria-Bertani (LB) broth or agar plates at 37°C. *V. harveyi *BB170 was grown in LB or AB medium [17]. Where required, antibiotics were used at the following concentrations: 30 μg/ml chloramphenicol (for *E. coli luxS *mutants), 10 μg/ml tetracycline (*S. aureus *Newman), and 100 μg/ml kanamycin (*V. harveyi *BB170 and BB886).

### Autoinducer bioassays

The *V. harveyi *BB170 bioassay was used for the detection of AI-2 activity in culture supernatants or *in vitro *reactions. Initially the procedure outlined by Bassler *et al*. [17] was followed. For more careful analyses of *luxS *mutants, the assay was modified so that the AB medium employed contained 25 mM HEPES buffer, pH7.8, as additional component. AI-2 activity was defined as the fold increase in light production in comparison with medium or buffer controls. *In vitro *reactions were also tested using the AI-1 responsive reporter *V. harveyi *BB886 [17].

### Generation of *luxS *mutants

The *luxS *deletion of *E. coli *Bl21 *luxS *[9] was transferred into *E. coli *MG1655 wildtype and an *E. coli *MG1655 *pgi*-*edd*-*eda *triple mutant (*E. coli *pgi-EDP, [25] by phage transduction using the bacteriophage P1 and a standard protocol [36]. Successful transfer of the locus was established by PCR, using the primer pair LuxS-50-UF (CTCAGACTCGCCTGGGAAGAAAGAG) and LuxS-50-DR (GTGCGCACTAAGTACAACTAAGCC).

### Preparation of sugar phosphates for the *in vitro *production of AI-2

Sugar phosphate solutions were prepared in 10 mM sodium phosphate buffer (pH 7.7) and, where indicated, were incubated at 37°C. Following the indicated incubation period each sample was frozen using dry ice and stored at -80°C. Rul-5-P and other sugar phosphates were obtained from Fluka/Riedel-de Haën and Sigma-Aldrich, respectively.

Enzymatic synthesis of Rul-5-P was carried out by incubation of 5 mM ribose-5-phosphate with 10 U/ml spinach phosphoriboisomerase in 10 mM sodium phosphate buffer (pH 7.7). The reaction was incubated at 30°C for 2 h. Catalysis was stopped by chloroform extraction of the enzyme followed by freezing the samples on dry ice. For negative controls, spinach phosphoriboisomerase was inactivated by heat treatment (5 min at 85°C).

### Determination of DPD and MHF

DPD was detected after its derivatisation with *o*-phenylenediamine as described by Hauck et al. [13]. Detection and quantification of MHF was achieved by high-performance liquid chromatography coupled to tandem mass spectrometry (HPLC-MS-MS) after chemical derivatisation of the compound as described by Husek [37].

## Abbreviations

AI-2: Autoinducer-2; DPD: 4,5-dihydroxy-2,3-pentanedione; LC-MS/MS: liquid chromatography/tandem mass spectrometry; MHF: 4-hydroxy-5-methyl-3(2H)-furanone; OPPP: oxidative pentosephosphate pathway; Rib-5-P: ribose-5-phosphate; Rul-5-P: D-ribulose-5-phosphate; *S*-THMF-borate: (2*S*,4*S*)-2-methyl-2,3,3,4-tetrahydroxytetrahydrofuran-borate; *R*-THMF: (2*R*,4*S*)-2-methyl-2,3,3,4-tetrahydroxytetrahydrofuran; Xyl-5-P: xylulose-5-phosphate.

## Authors' contributions

TJT performed the Rul-5-P *in vitro *experiments and AI-2 bioassays. He also generated the *luxS *mutants and performed the growth experiments. NMH carried out all chemical analyses, looking at DPD and MHF formation in both culture supernatants and in vitro reactions. KW conceived and designed this study and wrote the manuscript. KRH contributed to the design of this study and sections of the manuscript. All authors contributed to data analysis and interpretation.
